# Gene Expression Profiling for *In Silico* Microdissection of Hodgkin's Lymphoma Microenvironment and Identification of Prognostic Features

**DOI:** 10.1155/2011/485310

**Published:** 2010-12-13

**Authors:** François Bertucci, Bruno Chetaille, Luc Xerri

**Affiliations:** ^1^Department of Bio-Pathology, Institut Paoli-Calmettes, 232, bd Sainte-Marguerite, 13273 Marseille Cedex 09, France; ^2^Department of Medical Oncology, Institut Paoli-Calmettes, 13009 Marseille, France; ^3^Faculty of Medicine, University of Mediterranee, 13284 Marseille Cedex 07, France

## Abstract

Gene expression profiling studies based on DNA microarrays have demonstrated their ability to define the interaction pathways between neoplastic and nonmalignant stromal cells in cancer tissues. During the past ten years, a number of approaches including microdissection have tried to resolve the variability in DNA microarray measurements stemming from cancer tissue sample heterogeneity. Another approach, designated as virtual or *in silico* microdissection, avoids the laborious and time-consuming step of anatomic microdissection. It consists of confronting the gene expression profiles of complex tissue samples to those of cell lines representative of different cell lineages, different differentiation stages, or different signaling pathways. This strategy has been used in recent studies aiming to analyze microenvironment alterations using gene expression profiling of nonmicrodissected classical Hodgkin lymphoma tissues in order to generate new prognostic factors. These recent contributions are detailed and discussed in the present paper.

## 1. Introduction

Classical Hodgkin lymphoma (cHL) is a disease of relatively good prognosis with a current overall cure rate of about 80%. However, few significant progresses have been done during the last decades. Therapeutic decisions remain largely based upon Ann Arbor staging (limited versus advanced disease) and the International Prognostic Index, IPI [1], which applies only to advanced stages. About 35% of patients are refractory to initial treatment or relapse after achieving complete remission. Furthermore, many patients are overtreated with both radio- and chemotherapy because of the lack of markers that could reliably predict long-term survival. In this context, the identification of biologic markers that could help to more accurately select cHL patients at high risk of treatment failure and patients with low-risk disease remains a crucial challenge [2]. 

Classical HL lesions are characterized by the presence of a minority of malignant cells (usually <5%), designated as Reed-Sternberg (RS) cells, which reside in a complex and abundant mixture of reactive cells composed of T- and B-cells, macrophages, plasma cells, and granulocytes [3]. The frequency and distribution of these cell components differ considerably between patients and between histological subtypes of disease. Their different proportions likely explain the lack of clinical applications of molecular analyses reported during many years. However, advances in our understanding of the cHL pathophysiology are emerging from the analysis of this microenvironment. As suggested by the correlation between the clinical course of cHL patients and the plasma levels of particular cytokines [4], the severity of the disease may result from cell signaling networks operating within neoplastic tissues. Reactive cells are thought to favor the proliferation of RS cells through cytokines and chemokines acting as paracrine factors [5]. An aberrant immune response in the vicinity of RS cells is supposed to account for the maintenance of an immunosuppressive environment. It has been initially proposed that a local Th2 reaction predominates, whereas Th1 cells, CD8 cytotoxic T-cells and NK cells are lacking [6]. More recently, it was suggested that T-reg cells and PD1+ T-cells also interact with RS cells, which produce the T-reg attractant galectin and the PD-1 ligand, PDL-1 [7, 8]. On the other hand, the observation of numerous CXCR3+ lymphocytes in some cHL tumors has raised the possibility of an occasional Th1-predominant immune response [9].

The functional role of the microenvironment in the pathophysiology of cHL remains a matter of debate, specially regarding the role of Th2 and T-reg cells, which bear a paradoxically favorable prognostic value [10–12]. An accumulating number of immunohistochemistry (IHC) studies attempted to evaluate the composition and prognostic significance of tumor-infiltrating lymphocytes (TILs) subpopulations. More recently, gene expression profiling studies based on DNA microarrays have demonstrated their ability to more accurately define the interaction pathways of RS cells with nonmalignant reactive and stromal cells in lymphoma tissues. This is the scope of this paper.

## 2. DNA Microarray-Based Gene Expression Profiling and Microdissection

So far, DNA microarrays represent the most developed and used high-throughput molecular technique. They allow the simultaneous analysis of mRNA expression level of tens-of-thousands of genes in a single step, thus providing an actual molecular portrait of biological sample [13]. Potential applications are multiple, from a better understanding of oncogenesis to the improvement of diagnostic and prognostic classifications and the development of new anticancer drugs. Breast cancer has been so far one of the most extensively analyzed solid tumors with very promising results [14, 15].

**Figure 1:**
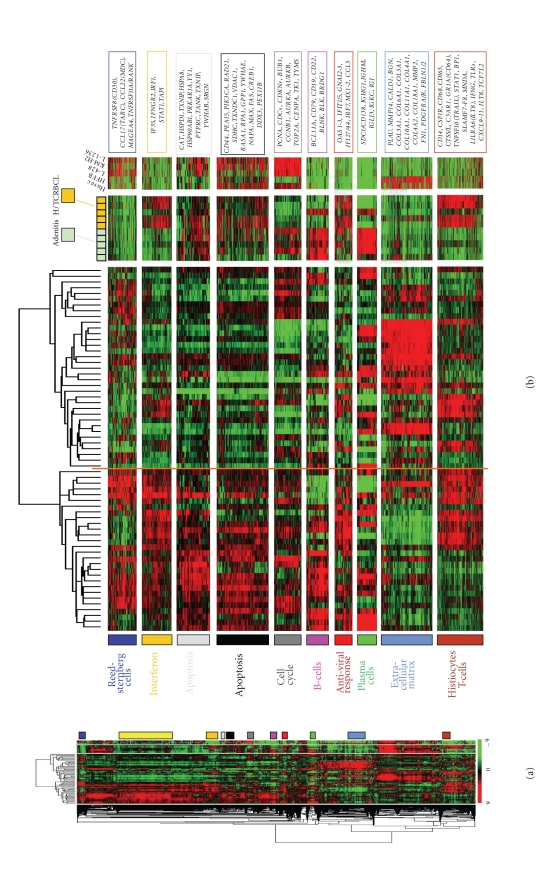
Global gene expression profiling of cHL samples and control samples.

During the past ten years, a number of approaches have tried to resolve the variability in DNA microarray measurements stemming from cancer tissue sample heterogeneity. This heterogeneity due to the surrounding reactive cell types can lead to the identification of differentially expressed genes that may be unrelated to any biological question regarding neoplastic cells. Anatomic microdissection is one possibility, which allows the procurement of pure cell subpopulations from frozen, fresh, or fixed tissues. This can be achieved for example by laser microdissection [16, 17] or by mechanical dispersion followed by flow cytometry. This later was successfully applied in colorectal cancers for characterization of the tumor-infiltrating immune cells by gene expression profiling [18]. Another approach designated as virtual or *in silico* microdissection [19] avoids the laborious and time-consuming step of anatomic microdissection. It consists in confronting the gene expression profiles of complex tissue samples to those of cell lines representative of different cell lineages, different differentiation stages, or different signaling pathways. This strategy was initially applied to derivate biologically relevant subtypes of tumors with different survival in breast cancer [20] and in B-cell non-Hodgkin lymphomas [21–24]. To date, numerous gene expression signatures of immune cells are available in databases like Signature DB [25, 26], and statistical methods, such as GSEA (Gene Set Enrichment Analysis) [27], can define whether any of them is differentially expressed in a subgroup of samples. Classical HL, with abundant stromal component, appears specially adapted to this approach. For instance, we have identified a “Reed Sternberg cell” cluster of 97 probe sets characterized by overexpression of genes like TNFRSF8 (CD30), CCL17 (TARC), CCL22 (MDC), MAGEA4, and TNFRSF11A (RANK) [28]. As expected, this cluster was strongly expressed in HL cell lines but was not expressed in non-Hodgkin's cell lines and tissues. This cluster and other clusters associated with biological processes and other cell types are shown in Figure 1. Figure 1(a) shows the Hierarchical clustering of 63 cHL tissue samples based on 6.229 probe sets with significant variation in mRNA expression levels across these samples. Each row represents a gene and each column represents a sample. The two separated color matrixes on the right side correspond to the expression profiles of control tissue samples (5 benign lymphadenitis, 5 H/TCRBCL samples, and 5 cell lines from left to right, resp.). Since these control samples are not considered in the clustering of cHL samples, genes are in the same order than in the major left matrix. The expression level of each gene in a single sample is relative to its median abundance across the 63 cHL tissue samples and is depicted according to a color scale (log_2_ scale) shown at the bottom. Red and green indicate expression levels, respectively, above and below the median. The magnitude of deviation from the median is represented by the color saturation. The dendrogram of cHL tissue samples (above matrix) represent overall similarities in gene expression profiles, whereas colored bars on the right side indicate the locations of 11 gene clusters of interest. Figure 1(b) is a Zoomed view of panel (a), highlighting the dendrogram and gene clusters. In the dendrogram (top) of cHL tissue samples, two large groups are evidenced by clustering and delimited by an orange vertical line. The control samples are color coded: green for lymphadenitis, orange for H/TCRBCL samples. The 5 cell lines are Huvec (endothelial cells), HFFB (fibroblastic cells), L-428, KM-H2, and L-1236 (Reed-Sternberg cells) (from left to right, resp.). Expanded view of selected gene clusters corresponding to relevant cell types/function are named from top to bottom: “Reed Sternberg cells” (dark blue bar), “interferon pathway and antiviral response (1)” (orange bar), “apoptosis” (two clusters; light grey and black bars), “cell cycle” (dark grey bar), “B-cells” (pink bar), “interferon pathway and antiviral response (2)” (red bar), “plasma cells” (green bar), “extra cellular matrix” (light blue bar), “histiocytes/T-cells/innate immune response” (brown bar). The “cell metabolism” cluster (yellow bar in Figure 1(a)) is not zoomed in Figure 1(b). The most relevant genes included in these clusters are indicated on the right by their *EntrezGene* symbol, adapted from [28].

A few recent studies, described in the following section, have demonstrated the feasibility of analyzing microenvironment alterations using gene expression profiling of nonmicrodissected cHL tissues in order to generate new prognostic factors.

## 3. Gene Expression Profiling of Nonmicrodissected cHL Tissues

To date, only four studies of gene expression profiling of cHL tissue samples have been published [28–31]. They have been reported by three teams, including ours. All used frozen nonmicrodissected pretreatment samples obtained from patients with cHL during diagnostic lymph-node biopsy. The number of profiled samples was relatively small, but the last three studies combined tissue microarray (TMA) analysis to validate the prognostic value of microenvironment alterations in large and independent series [28, 30, 31]. Different control samples were included through these studies: cell lines representing cell types found in the microenvironment (normal B- and T-cells, normal fibroblastic and endothelial cells), and RS cells, as well as tissue samples (adenitis, B- and T-cell lymphomas). The main characteristics of these studies are described in Table 1.

The first study was reported by our group in 2002 [29]. For the first time, it showed the feasibility of profiling cHL tissue samples with DNA microarrays, the transcriptional heterogeneity of samples, and already, it suggested the existence of correlations between gene expression profiles, notably genes of the microenvironment, and prognosis [29]. However, the number of genes was relatively small, the number of cases (*n* = 21) was too limited to draw a final conclusion, and no validation set was available.

The second study was published by the Spanish Hodgkin Lymphoma Study Group in 2006 [30]. Authors profiled 29 advanced stage cHL (14 patients with favorable outcome and 15 with unfavorable outcome), and found a 145-gene signature linked to poor response to treatment. These genes were grouped into four clusters representing genes expressed by either the tumor cells (regulation of mitosis and cell growth/apoptosis) or the tumor microenvironment. Importantly, the link with survival was validated for 8 genes by IHC in an independent validation set of 235 cHL samples spotted onto TMA. Finally, the authors validated at the functional level alterations in the regulation of the mitotic checkpoint in RS cells. Relative limitations of this study included the small number of cHL profiled using DNA microarrays, the selection of advanced stages only, and the number of genes (*n* = 9,348). 

Recently, we profiled cHL tissue samples collected from 63 patients with either localized or advanced cHL using whole-genome U133 A 2.0 Affymetrix microarrays [28]. Whole-genome clustering confirmed the molecular heterogeneity of cHL samples. By comparing the expression profiles of 31 cases with favorable outcome and 21 with unfavorable outcome, we identified a 450-gene list associated with survival. Genes related to B cells and apoptosis were related to good prognosis, whereas genes associated with stroma remodeling were related to poor prognosis. An independent set of 146 cHL samples was analyzed using IHC. We also reported a gene signature associated with the EBV status of samples (18 EBV+ versus 35 EBV−), which was characteristic of Th1 antiviral immune response. Finally, a 614-gene signature was generated by comparison of cHL samples (*n* = 63) and H/TCRBCL samples (*n* = 5). For both signatures, validation was obtained by IHC for some physiologically relevant genes. 

The most recent study, published by the British Columbian Cancer Agency on March 2011, is the greatest one in terms of number of samples and genes tested [31]. Expression profiles of cHL samples from 130 patients of all stages were obtained with the use of whole-genome U133 Plus 2.0 Affymetrix microarrays. The authors defined a gene signature of tumor-associated macrophages associated with primary treatment outcome. They validated the correlation in an independent cohort of 166 patients by IHC and TMA with a single marker of normal macrophages, CD68. 

Although these four studies reported prognostic signatures related to microenvironment cells, it is noteworthy that there is only limited overlap between the genes of the signatures, as observed for example in breast cancer [14]. Methodological and more conceptual reasons explain this discrepancy. There are many methodological differences between the four studies, related to patients and technologies used. In our studies and the American study, patients presented either localized or advanced disease, whereas all patients enrolled in the Spanish study presented with advanced disease [30]. Treatments and followup are also different. Different platforms of DNA microarrays were used, with different types of probes (cDNA clones, or oligonucleotides), with different RNA labeling methods (radioactivity, or fluorescence), and with different gene sets. Our last study and the American one [28, 31] used Affymetrix oligonucleotide microarrays containing, respectively, ~16,000 and ~25,000 genes, whereas the Spanish study used spotted microarrays (OncoChip.v2 cDNA microarrays produced at the CNIO) containing 11,675 human clones representing 9,348 genes selected on the basis of their proven or putative involvement in cancer [30]. Methods of data analysis are also different. More conceptually, cHL is a heterogeneous disease, and lists of discriminator genes are unstable, notably when they are generated from small sets of tumors and/or when the correlations between gene expression and outcome are limited. Another explanation is that discriminator genes, even if different among studies, are involved in the same pathways or cell processes. A classical example is breast cancer, for which different signatures carry similar information with regards to prognostication [32] because in fact driven by a common force, the cell proliferation [33]. The four cHL studies shared some signatures associated to similar cell types. Importantly, an independent validation of clinico-genomic correlations was present in the last three studies [28, 30, 31]. Authors reported the prognostic value of single promising markers by IHC analysis, which could be more easily used in clinical routine than DNA microarrays. Finally, multivariate analysis, which was performed only in the two last studies [28, 31], demonstrated the independent prognostic value of markers (BCL11A and CD68, resp.), which outperformed the conventional prognostic features.

## 4. Influence of B Cells

An unexpected finding stressed out by the 2 Affymetrix studies is the favorable role of reactive B cells within cHL tissues. We observed that many probe sets of a “B-cell” cluster, such as those encoding for BCL11A, BANK1, STAP1 (BRDG1), BLNK, FCER2, CD24, and CCL21, were associated with favorable outcome [28]. This led us to assess and confirm the prognostic influence of B cells using IHC with CD20 and BCL11A antibodies in a series of 146 patients. In multivariate analysis adjusted for classical prognostic factors, CD20 and BCL11A remained as informative parameters for overall survival (OS). Multivariate analysis of event-free survival (EFS) led to a final model including only BCL11A and leucocyte count [28]. BCL11A is a transcription factor expressed in normal pDCs and B cells, and in primary mediastinal B-cell lymphoma cells [34]. When we analyzed separately the pDC and B-cell populations using antibodies against BDCA2 and CD20, the amount of CD20+ reactive cells was correlated with better outcome, although less significantly than BCL11A, whereas the number of BDCA2+ pDCs showed no significance. 

Our findings were validated by the American study, which found that an increased number of CD20+ small B cells was associated with prolonged progression-free survival (PFS) and disease-specific survival (DSS) in univariate analysis [31]. However, the number of CD20+ small B cells strongly correlated with advanced stage disease and did not maintain any prognostic value in multivariate analysis.

Of note, the role of B cells was much less evident in the 2 other studies, although both noticed that the signature overexpressed by the favorable outcome group included molecules expressed by specific populations of B cells like IRTA2, VDR [30], and CD22 [29]. 

The association of high intratumoral B-cell counts with better outcome is in accordance with the excellent prognosis of the particular variant of cHL referred to as “nodular, lymphocyte-rich cHL” (NLRHL), which contains important B-cell amounts [35]. Since the histological type of our B-cell rich cHL cases was not NLRHL but either MC or NS, these cases could correspond to intermediate points on a putative spectrum spanning from NLRHL to NS or MC, depending on the B-cell content of lesions.

Of note, the correlation between the number of CD20+ B cells and survival in cHL patients appears paradoxical in the light of clinical studies showing encouraging results with the use of rituximab or anti-CD20 radio immunoconjugates [36, 37]. This paradox may be only apparent since the mechanism of rituximab activity is probably complex and may be related to the existence of circulating clonotypic B-cells potentially responsible for the generation of RS cells [38].

## 5. Influence of Macrophages

The first hint of the macrophage influence was given by the Spanish study [30]. Authors reported a signature overexpressed in the unfavorable outcome group of patients, which mainly included genes expressed by specific subpopulations of macrophages like *ALDH1A1*, *LYZ*, and *STAT1*. However, the clinical value of tumor-associated macrophages was not tested by IHC. Recently, Steidl et al. [31] confirmed that the overexpression of a macrophage signature was associated with the failure of primary treatment. Moreover, they validated this data using IHC. They found that an increased number of CD68+ cells in the diagnostic sample was associated with a poorer outcome in an independent set of 166 samples. An increased number of CD68+ macrophages correlated with a shortened PFS and with an increased likelihood of relapse after secondary treatment, notably autologous hematopoietic stem-cell transplantation, resulting in shortened DSS. In multivariate analysis, this adverse prognostic factor outperformed the IPS for DSS, but nor for PFS. The absence of an elevated number of CD68+ cells in patients with limited-stage disease defined a subgroup of patients with a long-term disease-specific survival of 100% with the use of current treatment strategies [31]. 

These results are in accordance with observations suggesting that a high number of macrophages correlates with inferior survival in follicular B-NHLs and in some epithelial cancers [39, 40]. To the extent that they have been investigated, tumor-associated macrophages have a phenotype and function similar to M2 macrophages, including poor cytotoxicity for tumor cells and promotion of tumor-cell proliferation induced by Th2 cytokines such as interleukin 4 (IL-4), IL-13, and IL-10 [41].

## 6. Influence of Inhibitory T-Cell Subsets

In our last gene profiling study of cHL, H/TCRBCL samples were included as controls because they represent the only lymphoma type that harbors an equivalent amount of reactive T cells and macrophages as found in HL [3]. Thus, the differences evidenced by gene expression profiling were expected to be mainly due to stromal components, rather than to the nature of neoplastic cells. This led to the identification of stromal markers that could differentiate cHL and H/TCRBCL samples. Genes overexpressed in H/TCRBCL belonged to functional categories including genes related to macrophages (*STAT1*, *LAMP1*) and Th1 response (*IFNG*, *CCR5*) [28]. One of the most significantly overrepresented genes in H/TCRBCL was *PDCD1/PD-1*, which codes for a lymphocyte inhibitory receptor expressed in specific lymphoma subtypes [42]. PD-1 protein expression was validated using IHC in 130 cHL and 13 H/TCRBCL. We showed that the majority of reactive T cells in H/TCRBCL expressed the PD-1 inhibitory receptor, whereas such PD-1+ T-cells were sparser in cHL. This implies that PD1 immunodetection on paraffin sections can be helpful for the differential diagnosis of H/TCRBCL versus HL, which may be sometimes difficult [43]. Sustained PD1 expression in H/TCRBCL may be induced by IFNG, whose transcripts were also more abundant in H/TCRBCL than in cHL tissues [28].

## 7. Influence of Other Stromal Components

In our pioneering study [29], we first reported that most genes overexpressed in the unfavorable cHL subset were also highly expressed in the HFF fibroblastic cell line, thereby suggesting a fibroblastic signature. These genes were related to fibroblast activation or function (*PDGFRB*, Collagen), but also to angiogenesis (Endostatin) or extra cellular matrix remodeling (*MFAP2*, *MMP2*, *MMP3*, *TIMP1*) [29]. More recently, we found in an independent set of cHL patients that genes of the “extra cellular matrix” such as collagen genes (*COL1A1/4A1/4A2/5A1/18A1*), *THBS1/2*, *FN1*, *EDNRA*, *ITGB5*, and *LAMA4* were associated with unfavorable outcome [28]. Of note, Steidl et al. reported MMP11, which encodes a matrix metallopeptidase, as associated with poor survival, both at the mRNA and protein levels [31].

By contrast, the Spanish study found that a set of genes overexpressed by the favorable outcome group was involved in adhesion and remodeling of the extra cellular matrix (*TIMP4*, *SPON1*, *LAMB1*) and in fibroblast function and chemotaxis (*TACR1*, *CCL26*) [30]. It is therefore possible that a subtle imbalance, rather than a mere increase, in the complex process of matrix remodeling plays a role in the aggressive behavior of HL tumors.

## 8. Influence of EBV on HL Microenvironment

EBV is present in RS cells of 40%–60% of cHL lesions and contributes to their pathogenesis [44, 45]. Immunologic reactions against EBV can occur in the peripheral blood of some cHL patients [44]. The intratumoral immunological alterations induced by EBV+ RS cells remain unclear. Before the use of gene profiling, only a few differences had been found between the microenvironment of EBV+ and EBV− RS cells, such as an increased expression of IP10/CXCL10 [9]. However, no comprehensive characterization had been reported. 

Gene profiling in search of EBV-induced alterations was first used in a series of 23 cHL cases [46]. Among these cases, EBNA1 was shown to up regulate the expression of CCL20 in EBV+ HL cells that in turn led to an increased chemotaxis of Tregs [46]. This mechanism might enable the escape of EBV-infected HL cells from the virus-specific CTL response.

We recently demonstrated that EBV+ and EBV− cHL tissues can be clearly separated from each other by a robust gene signature involving innate immunity and antiviral responses in EBV+ tumors [28]. In fact, the EBV+ cHL subset overexpressed antiviral genes like *IVNS1ABP* (NS1BP), *PLSCR1*, and *OAS*; together with the pattern recognition receptor TLR8 and the MDA5 helicase, which are both involved in the recognition of viruses of various structures [47]. Signaling through TLRs and helicases is known to converge toward induction of interferons, which are the principal cytokines mediating innate immunity against viral infection. The molecular profile of EBV+ tumors (simultaneous overexpression of *IFNG*, *CXCL9*, *CXCL10*, and *CXCL11/ITAC*) also provides evidence of intratumoral Th1 activity in EBV+ cHL [28]. This Th1 reaction could be orchestrated by IFNG, which is capable of inducing expression of not only *CXCL10*, but also *CXCL9/MIG* and *CXCL11/ITAC*, both known as CXCR3 ligands and potential chemoattractants of Th1 lymphocytes. Of note, *CXCL9* and *CXCL10* were also predominantly expressed in EBV+ cHL samples in the other gene profiling study, but without any evidence of Th1 or antiviral reaction [46]. This discrepancy may be due to the fact that the latter study was focused on cHL samples of the NS type only. It is noteworthy that our patients with EBV+ cHL did not have a better outcome, thereby suggesting that the described intratumoral immune reaction is inadequate to eliminate tumor cells. Nonetheless, these data raise the possibility that it could be further stimulated to design future therapies.

## 9. Conclusion

Despite the low number of gene profiling cHL studies available, they have led to dramatic results as regards the important influence of B cells and macrophages on patients' outcome. The resulting prognostic status can be evaluated in the routine diagnosis of cHL patients using IHC. Another interesting, although preliminary, attempt to translate gene profiling results into routine practice is to design a real-time PCR-based low-density array that includes the most relevant genes and that could be applied to formalin-fixed paraffin-embedded samples, as recently reported by the Spanish group [48, 49]. Besides, gene profiling turned out to be helpful in understanding the ability of EBV to either disable the CTL response or to trigger a Th1 reaction in the cHL microenvironment. This could be critical to the development of adoptive T-cell therapies that target the virus or different cell components of HL microenvironment. All these results are clinically promising. There is no doubt that their integration with results generated by other modern high-throughput molecular analyses [50–52] will further improve our understanding of disease, and likely the patients' management.

## Figures and Tables

**Table 1:** Gene expression profiling studies of nonmicrodissected cHL and survival.

Reference	cHL tumor samples	Microarray platform N° of genes	Control samples	Gene signature associated with	Validation set	Multivariate analysis
[29]	21 samples limited and advanced stages	Home-made cDNA ~ 1000 genes	Cell lines (RS and non RS) tissues (adenitis, lymphomas)	Sustained complete remission	No	No
[30]	29 samples advanced stages	Home-made (CNIO) cDNA ~ 9.348 genes	Cell lines (RS and non RS) tissues (adenitis)	Sustained complete remission	250 samples IHC TMA	No
[28]	63 samples limited and advanced stages	Affymetrix Oligonucleotides ~ 16.000 genes	Cell lines (RS and non RS) tissues (adenitis, lymphomas)	Sustained complete remission	146 samples IHC TMA	EFS OS
[31]	130 samples limited and advanced stages	Affymetrix Oligonucleotides ~ 25.000 genes	No	Absence of progression or relapse	166 samples IHC TMA	PFS DSS

cHL: classical Hodgkin lymphoma; RS: Reed Sternberg; IHC: immunohistochemistry; TMA: tissue microarray; EFS: event-free survival; OS: overall survival; PFS: progression-free survival; DSS: disease-specific survival.
